# Biological Characterization of *Cleome felina* L.f. Extracts for Phytochemical, Antimicrobial, and Hepatoprotective Activities in Wister Albino Rats

**DOI:** 10.3390/antibiotics12101506

**Published:** 2023-10-02

**Authors:** Heena Yaqub Shaikh, Shaik Kalimulla Niazi, Asmatanzeem Bepari, Mary Anne Wong Cordero, Shazima Sheereen, Syed Arif Hussain, Muthuraj Rudrappa, Shashiraj Kariyellappa Nagaraja, Shekappa Ningappa Agadi

**Affiliations:** 1 Department of P.G. Studies in Botany, Karnatak University, Dharwad 580003, Karnataka, India; heena.ks@kud.ac.in (H.Y.S.); rmuthuraj@kud.ac.in (M.R.); shashiraj@kud.ac.in (S.K.N.); 2 Department of Preparatory Health Sciences, Riyadh Elm University, Riyadh 12611, Saudi Arabia; kalimullaniazi@gmail.com; 3 Department of Basic Health Sciences, College of Medicine, Princess Nourah bint Abdulrahman University, Riyadh 11671, Saudi Arabia; ambepari@pnu.edu.sa (A.B.); macordero@pnu.edu.sa (M.A.W.C.); 4 Department of Pathology, Manipal Academy of Higher Education, Mangalore 576104, Karnataka, India; dr.shazimasheereen@gmail.com; 5 Respiratory Care Department, College of Applied Science, Almaarefa University, Diriyah, Riyadh 13713, Saudi Arabia; syhussain@mcst.edu.sa

**Keywords:** *Cleome felina*, phyochemicals, GCMS, antimicrobial, acute toxicity, hepatoprotective

## Abstract

The present study aims to explore the phytochemical constitution and biological activities of *Cleome felina* L.f. (Cleomaceae). *C. felina* (leaves, stem, and root) extracts (acetone, methanol, and water) were qualitatively assessed for phytochemical presence. Methanolic leaves extract revealed more positive phyto-compounds among all the extracts; further, methanolic leaves extract was evaluated for FTIR, EDX, GCMS, antimicrobial assay, acute toxicity, and paracetamol-induced hepatoprotective activity in Wister albino rats. FTIR and EDX analysis unveiled important functional groups and elements in the leaves. GCMS analysis of methanolic leaves extract exposed 12 active phyto-compounds: major constituents detected were 1-Butanol, 3-methyl-, formate-48.79%; 1-Decanol, 2-ethyl-13.40%; 1,6-Anhydro-β-d-talopyranose-12.49%; Ethene, 1,2-bis(methylthio)-7.22%; Decane-4.02%; 3-Methylene-7, 11-dimethyl-1-dodecene-3.085%; Amlexanox-2.50%; 1,2,3,4-Cyclopentanetetrol, (1α,2β,3β,4α)-2.07%; L-Cysteine S-sulfate-1.84%; n-Hexadecanoic acid-1.70%; and Flucarbazone-1.55%. The antimicrobial assay showed a moderate zone of inhibition against *S. aureus*, *B. cereus*, *E. coli*, *P. aeruginosa*, *C. albicans*, and *C. glabrata* at 100 µL/mL concentration. Additionally, acute toxicity revealed no behavioral sign of the toxic effect. The significant results were obtained for methanolic leaves extract (low-50 and high-100 mg/kg b.wt. dose) for hepatoprotective activity, where it dramatically reduced serum blood biochemical markers (AST, ALT, ALP, Total bilirubin, and cholesterol) and exhibited elevated hepatic antioxidant enzymes (SOD, CAT, and GSH) concentration with lipid peroxidation retardation. To conclude, *C. felina* methanolic leaves extract ameliorated important phytochemical compounds and showed significant antimicrobial and hepatoprotective efficacy; therefore, utilization of *C. felina* leaves suggested in pharmacological applications, and in numerous cosmetics, herbicides, and food industries, would be a great scope for future hepatoprotective drug designing.

## 1. Introduction

Plants are essential for human beings in their daily life; they play a vital role as primary and secondary metabolites by providing food as energy, fiber, shelter, phytonutrients, and beneficial health-promoting pharmaceutical applications. In accordance with the World Health Organization (WHO), herbal medicine is utilized as primary health care by about 80% of the global population [1]. The level of primary metabolites determines the nutritional or nutraceutical value of plants, constituting the composition of fat, carbohydrate, protein, fiber, vitamins, and minerals, whereas the level of secondary metabolites determines the medicinal and therapeutic values that have applications in pharmaceutical, food, cosmetics, and fine chemical industries [2]. In the present era, the use of natural plant-based products has increased in the preparation of medicine and nutritional products and in different health sectors, as plants have antibacterial, antifungal, antidiabetic, antioxidant, anti-hyperlipidemic, anti-hypercholesterolemic, analgesic, hepatoprotective, neuroprotective, anticancer, and anti-inflammatory activities [3,4]. Hence, the determination of chemical constituents of herbal plants is essential, which can be achieved by different analytical techniques, such as Fourier-transform infrared spectroscopy (FTIR), energy-dispersive X-ray spectroscopy (EDX), Gas Chromatography–Mass Spectroscopy (GC/MS), etc., which are used to determine functional groups, elemental composition, and identification of the phytochemical compounds in crude extract.

In the past few years, health services have faced problems, as pathogenic bacteria and fungi have adopted new resistance mechanisms to commercial drugs or antimicrobial agents. Hence, it is necessary to find alternate antimicrobial agents that have strong effects towards resistance, along with fewer side effects, which is possible by the discovery of herbal-based antimicrobial agents [5]. Moreover, the use of natural-based products has been recommended by the World Health Organization, but driven attention to their toxicity and safety while using the natural products [6].

On the other hand, synthetic or commercial drugs and xenobiotics act as a toxic agent to most human organs, mainly to the liver, which is one of the largest internal organs and plays a characteristic role in the regulation and synthesis of several metabolic, essential biochemical and physiological processes, which are involved in the fight against infections and the detoxification mechanism of xenobiotics, whereas, at the same time, it is the organ that is readily prone to hepatic injuries by hepatotoxic agents (ethanol, CCl4, thioacetamide, D-galactosamine, environmental toxins, paracetamol) that lead to changes in the physiology and functional mechanism of the liver, as well as later resulting in liver fibrosis and cirrhosis [7,8]. In the present scenario, paracetamol (PC), known as *N*-acetyl-p-aminophenol (acetaminophen), acts as an analgesic and antipyretic drug, and is highly consumed. Moreover, the overdose of PC drug by humans or experimental animals will result in an increase of oxidative stress that induces lipid peroxidation and causes toxicity to hepatic cells [9]. However, various endogenous resistance mechanisms will operate to scavenge ROS to limit undesired cellular damage, but this protection might not be complete; hence, the additional foreign resistance in the form of dietary antioxidants will help to cure excessive cellular damage that was caused by excessive formation of ROS [10,11]. Due to this, researchers are driving their focus to find the substitution herbal treatments with potent antioxidant activities, which exhibit fewer side effects for disease treatments, such as liver diseases [12].

The genus *Cleome* is reported to possess pharmacologically active phytocompounds, which have been isolated, as they perform a vital role in the treatment of several ailments. The various plant parts of the genus *Cleome* have revealed good nutritional and therapeutic value. The most important class of secondary phyto-compounds has been derived from the *Cleome* species, which includes terpenes, sterols, flavonoids, glucosinolates, indole alkaloids, and isothiocyanates, which are distributed in various *Cleome* species [13,14]. *Cleome felina* L.f. is one among the species of the genus *Cleome*, which is a medicinal plant and is endemic to peninsular India [15]. This annual hairy herb is 30–60 cm, with trifoliate leaves, corolla pink, and more than 50 stamens; seedpods with seeds are slightly longer than the pedicel [16]. In epistaxis, the fresh and dry parts of *C. felina* are prescribed, which are pounded in equal quantity and given with milk and sugar. The complete plant is used as an astringent, and the vesicant seeds are given internally as a vermifuge [17]. Further, Wollenweber et al. [18] reported the isolation and characterization of flavonols, namely, 5,3′,4′-triOH-3,6,7,5′-tetraOMe flavone and 5,3′-diOH-3,6,7,4′,5′-pentaOMe-flavone (2), in *C. felina*. To date, several *Cleome* species have been studied for their active phytocompounds and pharmacological applications, but there is a dearth of information on *C. felina*. Hence, the objective of the current study was to screen *C. felina* for phytochemical constituents and to analyze pharmacological applications, such as antimicrobial activity, acute toxicity, and paracetamol-induced hepatoprotective activity, in Wister albino rats. The results of the investigation could provide scientific evidence of *C. felina* for its contribution in the therapeutic field.

## 2. Results

### 2.1. The Percentage Yield of Different Solvent Extracts of C. felina

**Figure 1:**
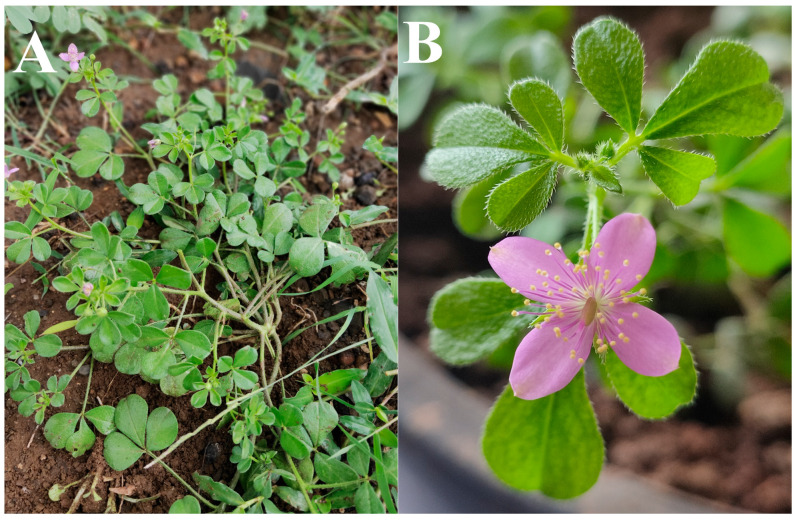
*Cleome felina* plant: (**A**) Plant habitat, (**B**) Plant with flower.

*C. felina* is a hairy herb with trifoliate leaves (Figure 1). It was collected, and each part was separated; it was then washed and dried. The coarse powder was obtained from the dried part and processed for solvent extraction using organic solvents by Soxhlet apparatus to obtain the extraction yield.

The percentage yield of the acetone, methanol, and water extract of *C. felina* leaves, stem, and root is displayed in Table 1. The leaves extracts of acetone, methanol, and water were 3.65, 17.01, and 13.75 g/100 g, respectively. The estimated amount of acetone, methanol, and water extract of the stem was 1.77, 9.63, and 15.64 g/100 g, respectively, whereas the extracts yield of acetone, methanol, and water for the root account for 1.4, 8.4, and 12.3 g/100 g, respectively.

### 2.2. Preliminary Screening of Phytocompounds for Different Solvent Extracts of C. felina

The various parts of *C. felina*, such as leaves, stem, and root, were extracted using organic solvents (acetone, methanol, and water). Each selective plant part with its chosen solvent was qualitatively analyzed for the presence of phytochemical constituents, such as carbohydrates, amino acids, glycosides, cardiac glycoside, phenolic, flavonoids, saponins, terpenoids, anthraquinones glycosides, and quinones, present in it for the screening of better solvent extracts to be used for further research work (Table 2). The phyto-compounds screening of leaves extract was estimated to show the presence of 6 acetone, 12 methanol, and 9 water metabolites. On the other hand, stem extract showed the presence of 10 acetone, 10 methanol, and 8 water phytochemicals, while the study of root extract exhibited the presence of 5 acetone, 9 methanol, and 6 water plant metabolites.

### 2.3. Fourier-Transform Infrared Spectroscopy (FT-IR) and Energy-Dispersive X-ray Spectroscopy (EDX) Analysis

**Figure 2:**
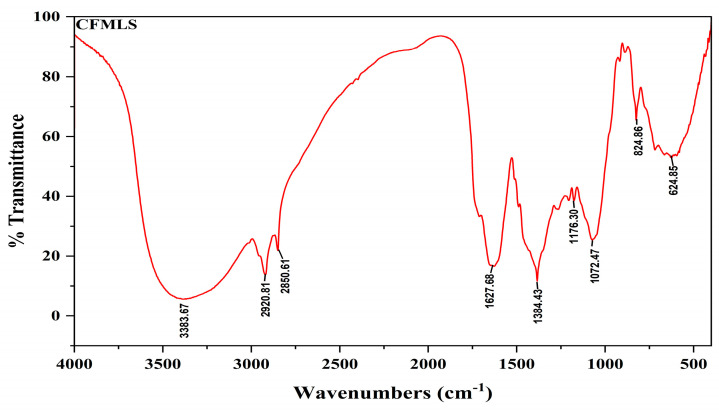
FT-IR chromatogram showing peak values for bioactive functional groups of *C. felina* methanolic leaves extract.

**Figure 3:**
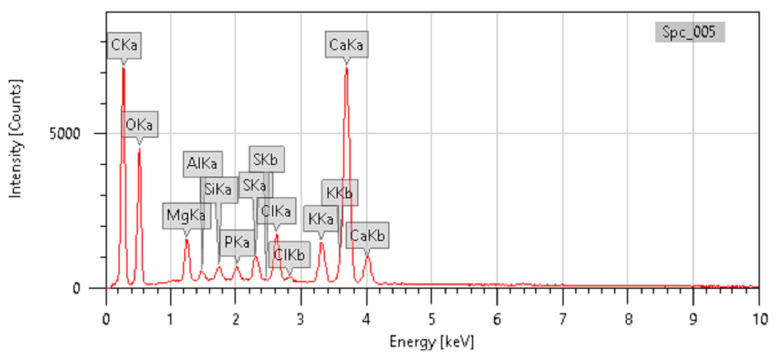
EDX chromatogram of *C. felina* leaves indicating elemental peaks.

The IR spectrum for methanolic extract of the *C. felina* leaves was obtained by a NICOLET 67000 FTIR Spectrophotometer (NICOLET, Thermo Fisher Scientific, Waltham, MA, USA), and the transmittance of FT-IR analysis was recorded under the range of the 400–4000 cm^−1^ IR region. Wavenumber (cm^−1^) intensities for a functional group and compound class were documented as presented in Figure 2 and Table 3. For the absorption spectrum of the methanolic leaves extract, the main peak showed the occurrence of various classes of compounds, such as alcohols, amine salt, conjugated alkenes, sulfonyl chloride, and halo compounds. Major bands were observed at 3383.67 cm^−1^, 2920.81 cm^−1^, 2850.61 cm^−1^, 1384.43 cm^−1^, 1176.30 cm^−1^, and 824.86 cm^−1^ due to the vibration of O-H, N-H, S=O, C-O, C-Cl, and C-Br, respectively. Meanwhile, the mineral composition of *C. felina* leaves was analyzed by the energy-dispersive X-ray spectroscopy (EDX) technique with respect to the mass % and is presented in Figure 3 and Table 4. The minerals found in leaves were carbon, oxygen, magnesium, aluminum, silicon, phosphorus, sulfur, chlorine, potassium, and calcium of 36.48, 39.74, 1.80, 0.35, 0.46, 0.58, 1.01, 2.15, 2.31, and 15.13 mass %, respectively.

### 2.4. Phytochemical Determination of C. felina Methanolic Leaves Extract by Gas Chromatography–Mass Spectrometry (GCMS) Analysis

**Figure 4:**
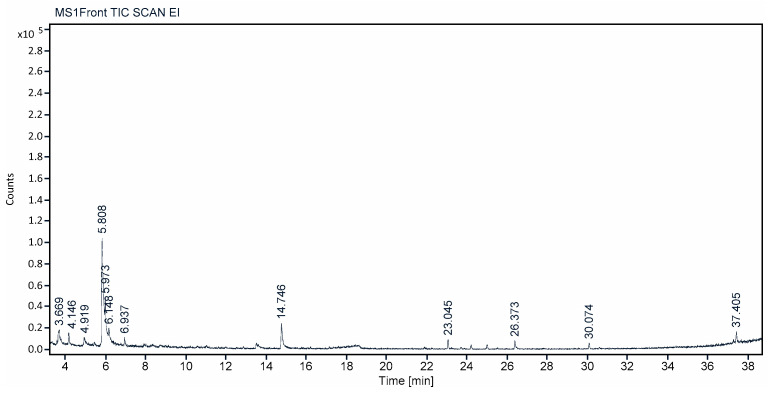
GCMS chromatogram showing peak values for volatile phytochemicals of *C. felina* methanolic leaves extract.

The methanolic leaves extract of *C. felina* were investigated for volatile phytochemical composition by GCMS analysis and were confirmed by NIST library mass spectrum. In total, 12 different phyto-compounds were identified in the leaves, and methanolic extract are of important applications. The retention time, peak area, area percent, name of the compound, molecular formula, and molecular weight are separately depicted for each identified phyto-compound in Figure 4 and Table 5. The phyto-constituents with high-percent composition were found to be 1-Butanol, 3-methyl-, formate (48.79%); 1-Decanol, 2-ethyl- (13.40%); 1,6-Anhydro-β-d-talopyranose (12.49%); Ethene, 1,2-bis(methylthio)- (7.22%); and Decane (4.02%), while considerable-percent composition of phyto-compounds detected were 3-Methylene-7, 11-dimethyl-1-dodecene (3.085); Amlexanox (2.50%); 1,2,3,4-Cyclopentanetetrol, (1α,2β,3β,4α) (2.07%), L-Cysteine S-sulfate (1.84%); n-Hexadecanoic acid (1.70%); Flucarbazone (1.55%); and 4-Bromo-2, 6-difluorobenzyl alcohol (1.35%). Each identified compound is specific for biological activities and is a specific role of metabolite.

### 2.5. Antimicrobial Activity of C. felina Methanolic Leaves Extract

**Figure 5:**
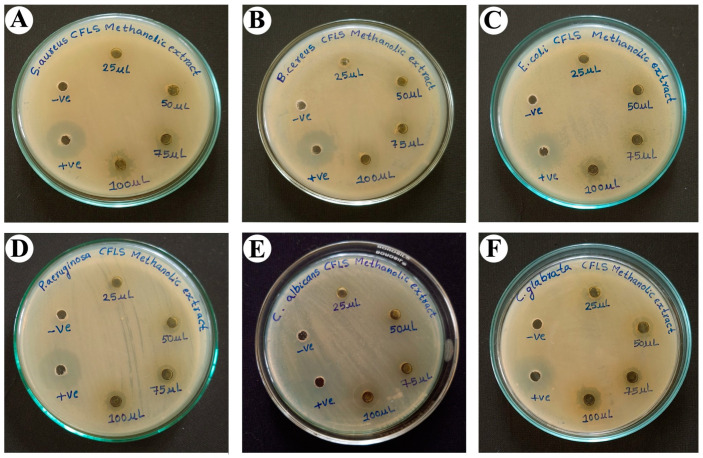
Antimicrobial activity of methanolic leaves extract of *C. felina* showing zone of inhibition at different concentrations against (**A**) *S. aureus*, (**B**) *B. cereus*, (**C**) *E. coli*, (**D**) *P. aeruginosa*, (**E**) *C. albicans*, and (**F**) *C. glabrata*.

Antimicrobial activity for two positive *Staphylococcus aureus* (*S. aureus*) and *Bacillus cereus* (*B. cereus*) bacteria, two negative *Escherichia coli* (*E. coli*) and *Pseudomonas aeruginosa* (*P. aeruginosa*) bacteria, and two yeast *Candida albicans* (*C. albicans*) and *Candida glabrata* (*C. glabrata*) strains was studied by the agar well diffusion method for *C. felina* methanolic leaves extract at 25, 50, 75, and 100 µL/mL concentrations, depicted in Figure 5 and Table 6, wherein streptomycin and nystatin were used as the positive control and DMSO as the negative control. The observations for the formation of the inhibition zone by methanolic leaves extract were made, which implies that methanolic leaves extract was found to show a potential zone of inhibition against *S. aureus*-12.3 ± 7.84 mm, *B. cereus*-13.67 ± 8.69 mm, *E. coli*-11.33 ± 7.21 mm, *P. aeruginosa*-10.67 ± 6.78 mm, *C. albicans*-14.67 ± 9.33 mm, and *C. glabrata*-12.33 ± 7.84 mm at a 100 µL/mL concentration, which was comparable to the positive control, while the moderate zone of inhibition was observed at a 75 µL/mL concentration against *S. aureus*-8.67 ± 5.51 mm, *B. cereus*-10.67 ± 6.78 mm, *E. coli*-7.67 ± 4.87 mm, *P. aeruginosa*-7.67 ± 4.87 mm, *C. albicans*-11.67 ± 7.42 mm, and *C. glabrata*-7.33 ± 4.66 mm; further, *P. aeruginosa*-5.33 ± 3.39 mm and *C. glabrata*-5.67 ± 3.60 mm were also found to be sensitive at a 50 µL/mL concentration, whereas all selected pathogens showed resistance at a 25 µL/mL concentration, except *C. glabrata*-3.33 ± 2.12 mm.

### 2.6. Acute Toxicity of C. felina Methanolic Leaves Extract

The acute toxicity for the methanolic leaves extract of *C. felina* was evaluated according to Organization for Economic Co-operation and Development (OECD) guidelines 425 by oral administration of the single-extract dose at concentrations of 84, 80, and 70 mg to the body weight (42, 40, and 35 g, respectively) of Swiss albino mice. The limit dose given per oral administration was 2000 mg/kg b.wt. The dosed animals were under uninterrupted observation for 4 h for gross behavioral and toxicological signs; they were further monitored for the next 14 days. From the results, it was interpreted that no such behavioral and toxicological signs were manifested, which implies that the selected dose of methanolic leaves extract was nontoxic and showed no side effects, as death did not occur in the treated animals.

### 2.7. Paracetamol-Induced Hepatoprotective Activity by C. felina Methanolic Leaves Extract in Wister Albino Rats

#### 2.7.1. Estimation of Serum Blood Biochemical Markers

**Figure 6:**
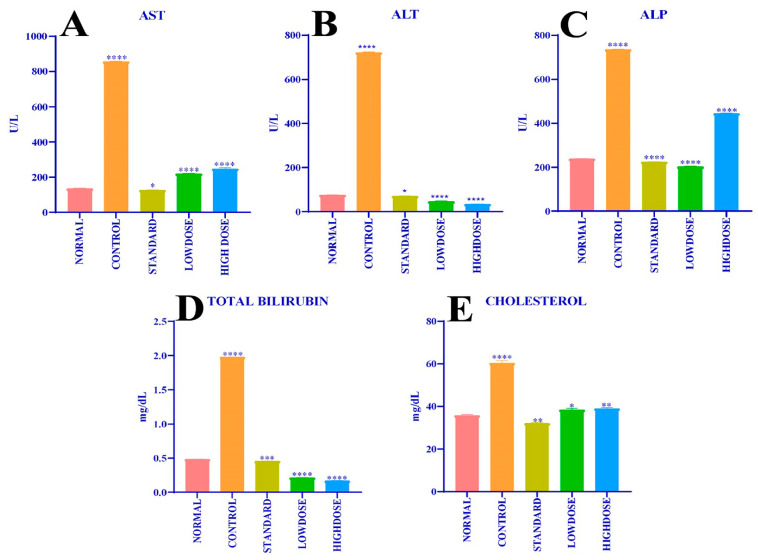
(**A**) Effect of different doses of CFMLS methanolic extract on AST level; (**B**) Effect of different doses of CFMLS methanolic extract on ALT level; (**C**) Effect of different doses of CFMLS extract on ALP level; (**D**) Effect of different doses of CFMLS methanol extract on total bilirubin level; and (**E**) Effect of different doses of CFMLS methanol extract on cholesterol level. All values presented as mean ± SEM, one-way Analysis of Variance (ANOVA), followed by multiple Dunnett’s test, * *p* < 0.05, ** *p* < 0.01, *** *p* < 0.001, and **** *p* < 0.0001 compared with normal group. (CFMLS: *C. felina* methanolic leaves extract).

Early hepatic damage is measured by the estimation of biochemical markers, such as aspartate aminotransferase-AST (Figure 6A), alanine aminotransferase-ALT (Figure 6B), alkaline phosphatase -ALP (Figure 6C), total bilirubin (Figure 6D), and total cholesterol (Figure 6E), present in the serum blood of experimental animals at the end of the practical day, presented in Figure 6A–E and Table 7. The conducted study showed that group 2 rats (control) constituted blood serum AST (857.7 ± 2.67 U/L), ALT (722.9 ± 3.20 U/L), ALP (736.9 ± 0.67 U/L), total bilirubin (1.98 ± 0.00 mg/dL), and total cholesterol (60.50 ± 1.12 mg/dL). In comparison, group 1 (normal) animal models of AST (136.2 ± 0.40 U/L), ALT (76.20 ± 0.48 U/L), ALP (238.1 ± 1.19 U/L), total bilirubin (0.48 ± 0.003 mg/dL), and total cholesterol (35.77 ± 0.38 mg/dL) was detrimental to group 1 animal serum, whereas group 3 (standard) rats’ blood serum AST (127.3 ± 0.91 U/L), ALT (70.85 ± 0.85 U/L), ALP (224.0 ± 0.49 U/L), total bilirubin (0.45 ± 0.001 mg/dL), and total cholesterol (33.13 ± 0.13 mg /dl) was minimal. Paracetamol-induced hepatoprotective activity by methanolic leaves extract of *C. felina* of group 4 (low dose-50 mg/kg b.wt.) rats with AST (220.5 ± 0.79 U/L), ALT (47.18 ± 0.82 U/L), ALP (203.1 ± 2.38 U/L), total bilirubin (0.21 ± 0.00 mg /dl), and total cholesterol (38.46 ± 0.67 mg /dl) was significant; similarly, the animals of group 5 (high dose-100 mg/kg b.wt.) in blood serum AST (248.0 ± 5.30 U/L), ALT (34.48 ± 0.48 U/L), ALP (445.0 ± 1.45 U/L), total bilirubin (0.17 ± 0.002 mg/dL), and total cholesterol (39.03 ± 0.41 mg/dL) were in considerable concentrations.

#### 2.7.2. Assessment of Liver Antioxidant Potential

**Figure 7:**
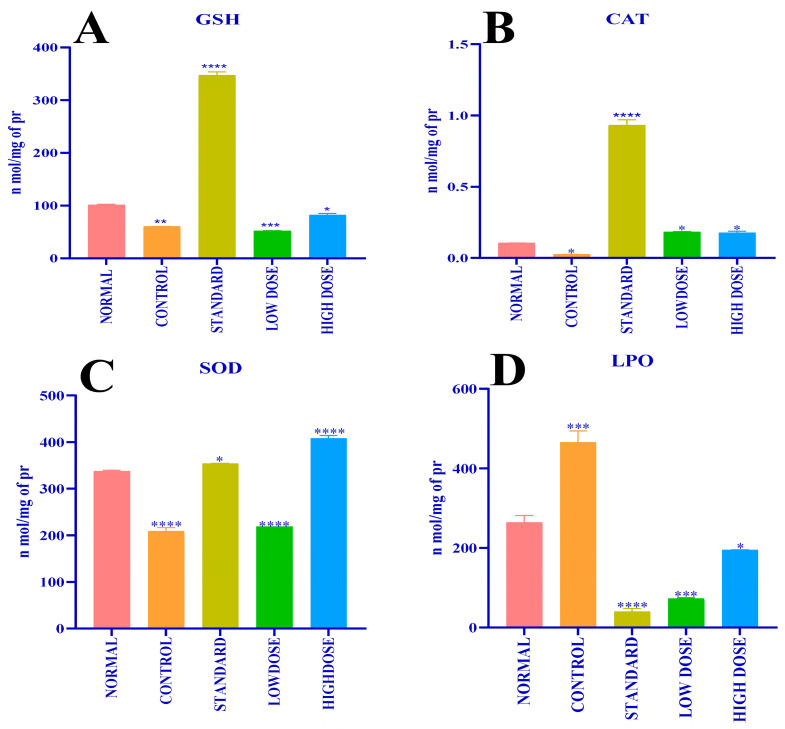
(**A**) Effect of different doses of CFMLS extract on Glutathione level; (**B**) Effect of different doses of CFMLS extract on Catalase level; (**C**) Effect of different doses of CFMLS methanol extract on SOD level; and (**D**) Effect of different doses of CFMLS methanol extract on Lipid peroxidation level. All values presented as mean ± SEM, one-way Analysis of Variance (ANOVA), followed by multiple Dunnett’s test, * *p* < 0.05, ** *p* < 0.01, *** *p* < 0.001, and **** *p* < 0.0001 compared with normal group. (CFMLS: *C. felina* methanolic leaves extract).

The methanolic leaves extract effects on the concentration of antioxidant enzymes, such as glutathione-GSH (Figure 7A), catalase-CAT (Figure 7B), superoxide dismutase (SOD (Figure 7C)), and level of lipid peroxidation-LPO (Figure 7D), in hepatic homogenate of experimental rats were analyzed and are displayed in Figure 7A–D and Table 8. Paracetamol-treated group 2 (control) rats exhibited a decline in the concentration of hepatic GSH (59.93 ± 0.30 n mol/mg of protein), CAT (0.02 ± 0.001 n mol/mg of protein), and SOD (208.9 ± 7.57 n mol/mg of protein) level and an increase in the LPO (465.7 ± 28.54 n mol/mg of protein) process; in relation to this, group 1 (normal) untreated PC animals showed an increased hepatic GSH (100.8 ± 1.53 n mol/mg of protein), CAT (0.10 ± 0.00 n mol/mg of protein), and SOD (337.2 ± 2.47 n mol/mg of protein) level and a decreased LPO (264.0 ± 17.48 n mol/mg of protein) reaction. The estimated amount of hepatic GSH (346.4 ± 6.77 n mol/mg of protein), CAT (0.93 ± 0.04 n mol/mg of protein), SOD (353.9 ± 0.58 n mol/mg of protein) level and LPO (40.00 ± 8.13 n mol/mg of protein) was significant in group 3 (standard) rats treated with silymarin. Methanolic leaves extract effects in group 4 (low dose-50 mg/kg b.wt.) and group 5 (high dose-100 mg/kg b.wt.) experimental rats were determined, where, in group 4 rats, the estimated concentration of hepatic GSH (51.29 ± 1.05 n mol/mg of protein), CAT (0.18 ± 0.003 n mol/mg of protein), and SOD (218.5 ± 0.00 n mol/mg of protein) antioxidant enzymes and LPO (72.65 ± 2.41 n mol/mg of protein) was significant. Meanwhile, group 5 rats’ antioxidant enzymes level for hepatic GSH (81.50 ± 3.80 n mol/mg of protein), CAT (0.17 ± 0.1 n mol/mg of protein), and SOD (407.9 ± 6.16 n mol/mg of protein) concentration and LPO (194.0 ± 0.59 n mol/mg of protein) reaction was as mentioned.

## 3. Discussion

Natural plant-based products are rich pockets of minerals and nutrients and have essential phyto-compounds with therapeutic properties. Worldwide, about 25% of prescribed drugs are obtained from plants, where, at present, about 121 active phyto-compounds are in use. The World Health Organization (WHO) has emphasized the basics and essentials of the 252 drugs; while 11% are tremendous of plant origin, polyphenols play a pivotal role in the maintenance of cellular antioxidant status by reducing free radicals, which inactivates inflammatory cytokines and production of chemokines and, as well, minimizes the generation of reactive oxygen species (ROS) and lipid peroxidation [19,20]. The extraction yield varies in accordance with the purity of the crude compound and the polarity of the solvent [21]. The present results of *C. felina* revealed that the leaves methanolic (17.01 g/100 g) extract yield was higher than the other solvent extracts. The high methanolic extract yield represents the occurrence of major polar chemical groups, such as phenols, flavonoids, alkaloids, steroids, glycosides, and other secondary metabolites [21].

Among the other parts of the *C. felina*, the methanolic leaves extract exhibited a maximum number of phytochemical compounds, such as carbohydrates, amino acids, glycosides, cardiac glycoside, phenolic, flavonoids, saponins, terpenoids, anthraquinones glycosides, and quinones. The closest abundance of phyto-constituents was noticed in the leaves (water) and stem (acetone and methanolic) extracts. The presence of bioactive compounds in the different parts of *C. felina* contributes to the therapeutic values of the plants in the treatment of diseases. In contrast to this, previously, several *Cleome* species were studied for the existence of phytochemical compounds in *C. spinosa* leaves and root extracts of cyclohexane, chloroform, ethyl acetate, and methanolic solvents [22]; *C. rutidosperma* leaves to water and ethanolic extracts [23], *C. gynandra* methanolic leaves extract [24], and *C. arabica* whole-plant aqueous-methanol, butanol, and water extracts [25], and it was reported the presence of flavonoids, saponins, terpenoids, anthraquinones, cardiac glycosides, tannins, carbohydrates, amino acids, proteins, polyphenols, reducing sugars, and alkaloids. The early preliminary findings reported by Silva et al. [22] and Alkhatib et al. [25] have determined that among comparative analyses between the different solvent extracts, the methanolic extract exhibited stronger positivity towards active phytochemical constitution than the other selective solvents during their studies.

The FTIR absorption spectrum of *C. felina* methanolic leaves extract revealed strong peaks at 3383.67 cm^−1^, 2920.81 cm^−1^, 2850.61 cm^−1^, 1384.43 cm^−1^, 1176.30 cm^−1^, and 824.86 cm^−1^, respectively, for the occurrence of alcohols, amines, conjugated alkenes, sulfonyl chloride, and halo compounds. The identified functional group contributes to the structural configuration of important compounds that are involved in different pharmacological activities and biosynthesis of functional metabolites. Similarly, Pillai and Nair [26] reported that whole-plant methanolic extract of *C. viscosa* and *C. burmanni* revealed the presence of phenols, alkanes, aldehydes, ketones, amines, amides, alkenes, carboxylic acids, sulfur compounds, alcohols, alkynes, and alkyl halides groups, while reports by Lingegowda et al. [27] on FT-IR analysis of methanolic leaves extract of *C. gynandra* exhibited the functional groups for important classes of compounds, i.e., phenols, amines and amides, carboxyl acid, silicon compounds, ketones, alkyl ketone, alkyl amine, alkanes, the alcohol hydroxyl group, and alkyl halides. Also, EDX analysis of *C. felina* methanolic leaves extract reports the presence of carbon, oxygen, sodium, magnesium, aluminum, silicon, phosphorus, sulfur, chlorine, potassium, and calcium elements. Similarly, Singh et al. [28] reported EDX analysis of *C. viscosa* seeds and confirmed the occurrence of carbon and oxygen, whereas a trace amount of magnesium, aluminum, silicon, sulfur, and calcium was observed. Elemental composition contributes to nutritional value and is proven for specific functions in metabolic activities and therapeutic values.

The current study on GCMS analysis of *C. felina* methanolic leaves extract exhibited 12 different phyto-compounds, in that the compound 1-Butanol,3-methyl-, formate, also known as isoamyl formate, is a short-chain ester possessing an aroma property, whose odor smells like pineapple, pear, and strawberry. Hence, it is utilized for flavoring and to intensify the flavor odor [29]. Earlier, Al-Dalali et al. [30] reported for 1-Butanol, 3-methyl-, formate, which is detected by GCMS analysis from vinegar, the compound 1-Decanol, 2-ethyl, which is alcoholic in nature, and 1,6-Anhydro-β-d-talopyranose is an exopolysaccharide. Ibrahim et al. [31] screened the 3% sulfuric acid leaves extract of *Gmelina arborea* (Verbenaceae family) for phyto-compounds by GCMS, in which 1,6-Anhydro-β-d-talopyranose was one among the other compounds detected, as well as it was reported that this compound acts as a toasted oak wood indicator and is used during wines ageing and distillates. The other compounds detected in *C. felina* methanolic leaves extract were Ethene, 1,2-bis(methylthio)-, Decane, 3-Methylene-7,11-dimethyl-1-dodecene, Amlexanox 1,2,3,4-Cyclopentanetetrol, (1α,2β,3β,4α) (carbohydrates), L-Cysteine S-sulfate, n-Hexadecanoic acid, Flucarbazone, and 4-Bromo-2,6-difluorobenzyl alcohol. The occurrence of Decane in the 80% ethanol extract of the *Cleome amblyocarpa* Barr. and Murb (stem) was reported by Khlifi et al. [32], estimated by GCMS analysis. The Amlexanox (azoxanthone drug) was used for curing mouth aphthous ulcers as a 5% topical oral paste and is the one approved clinically by the US FDA for aphthous ulcers treatment. It also exhibits novel applications in pharmacy [33,34,35,36]. L-Cysteine S-sulfate is an organic thiosulfate. It is an important class of L-cysteine molecule, which is S-substituted with a specified sulfur group. It is present as a plant and human metabolite and is involved in post-translation modifications, metabolisms, and pharmaceutical synthesis. In addition, it has detrimental effects on processes of cellular adhesive and tissue dehydration and has a role in the inactivation and clearance of small biomolecules of plasma [37,38]. n-Hexadecanoic acid is a saturated fatty acid. Most of the fatty acids are popular to possess antibacterial and antifungal activities. n-Hexadecanoic acid is highly studied and found to exhibit natural antioxidant, anti-inflammatory, antibacterial, anti-androgenic flavor, hypocholesterolemic nematicide, 5-Alpha reductase inhibitor, pesticide, hemolytic, and potent mosquito larvicide properties [39,40,41]. Perumal et al. [42] has reported the presence of n-Hexadecanoic acid in the *C. viscose* whole-plant ethyl extract analyzed by GCMS, species of the Cleomaceae family. Flucarbazone, previously called Flucarbazone-sodium, is a sulfonylurea herbicide. This herbicide is used to control dicotyledon weeds, such as Little seed Canarygrass, wild oats, and green foxtail, and reduces bermudagrass clippings grown as a weed during wheat and durum farming. However, it has been registered as an herbicide and growth regulator of turf grasses by U.S registration [43,44,45].

The evaluation of the antimicrobial assay of methanolic leaves extract has shown a significant inhibition zone against *S. aureus*, *B. cereus*, *E. coli*, *P. aeruginosa*, *C. albicans*, and *C. glabrata* at a 100 µL/mL concentration. Similarly, Weli et al. [46] reported the antimicrobial activity of *Cleome austroarabica* methanolic and different organic solvent extracts against *E. coli*, *H. influenza*, *E. faecalis*, and *S. aureus*, which did not show a zone of inhibition at selected concentrations. Another study conducted by Ghosh et al. [23] for antimicrobial activity of water and 70% ethanolic extract of *C. arabica* leaves against *S. aureus*, *E. coli*, and *V. cholerae*, individually, exhibited a moderate zone of inhibition. Khlifi et al. [32] performed the antimicrobial activity of *C. amblyocarpa* leaves and stem hydroalcoholic extract against four Gram-positive, four Gram-negative, and four yeast microorganisms using agar diffusion and micro-dilution methods. The growth inhibition of the entire tested microorganism at a dose-dependent manner was observed, and *E. coli* was found to be more sensitive among all.

The harmful side effects of the remedial substances were determined within 14 days by administrating the single-dose via oral routes and are carried to determine the median lethal dose (LD_50_) of specific toxic substances in experimental rats or mice [47]. The results of acute toxicity of *C. felina* methanolic leaves extract were non-toxic at the lethal dose of 2000 mg/kg b.wt. on single oral dose administration in Swiss albino mice. Previously, del Carmen et al. [48] analyzed the acute oral toxicity of dichloromethane ethanol and methanol at 0.5, 1, and 2 g/kg b.wt., hexane, and dichloromethane at 2 g/kg b.wt. extract of *Cleoserrata serrata* (Jacq.) Iltis. (Syn. *Cleome serrata* Jacq.) observed for the gain of body weight and alteration of organs (spleen, liver, kidney). Changes were not noticed in the body weight and organs of experimental rats examined at the microscopic level. Similarly, hydroalcoholic extract of *C. amblyocarpa* leaves and stem showed no mortality rate and no behavioral changes studied for the oral acute toxicity at 0.5, 1, and 3 g/kg b.wt. [32].

The current paracetamol-induced hepatoprotective study revealed that *C. felina* methanolic leaves extract at a low dose of 50 mg/kg b.wt. (group 4) and high dose-100 mg/kg b.wt. (group 5) remarkably deduced the serum blood biochemical markers for AST, ALT, ALP, total bilirubin, and total cholesterol. In the study, it was found that the level of serum blood biomarkers at the low dose-50 mg/kg b.wt. was significant when compared to the high dose-100 mg/kg b.wt. The significant reduction in the serum blood biomarkers by *C. felina* methanolic leaves extract was attributed to the healing ability of hepatic parenchymal tissues and hepatocytes. In contrast to this, Begum and Kiran [49] demonstrated the hepatoprotective activity of whole-plant methanolic extract of *C. chelidonii* induced by paracetamol and ethanol to estimate serum biomolecule markers. The 100, 200, and 400 mg/kg b.wt. doses were found to reduce the serum ALP, SGPT, SGOT, and TBIL levels.

The present study implies the in vivo antioxidant potential of *C. felina* methanolic leaves extract for hepatoprotective activity. The results of the experiment depicted that the methanolic leaves extract at the low dose-50 mg/kg b.wt. (group 4) and high dose-100 mg/kg b.wt. (group 5) ameliorated significant elevation of hepatic antioxidant enzymes with LPO retardation. Results of the conducted study indicate that the antioxidant enzyme estimation of hepatic tissue treated with *C. felina* methanolic leaves extract has exhibited similar results as that of the serum blood biochemical marker estimation, wherein the rats treated with a low extract concentration exhibited stronger effectiveness towards the hepatic oxidation than that of the animals treated with a high extract concentration. The significant elevation of antioxidant enzymes, such as GSH, CAT, and SOD, in rats treated with the methanolic leaves extract is the result restoration of metabolic enzymes that have been reduced due to endogenous oxidative stress induced by paracetamol. In relation to this, Singh et al. [14], in their review article, described the hepatoprotective activity of *C. viscosa* (leaves ethanolic extract, isolated coumarinolignoids from seeds, and seeds), administrated in a dose-dependent manner, and was equally significant to the standard drug Silymarin induced by CCl_4_. The reason of hepatoprotective activity might be due to the presence of polyphenols and flavonoids that have strong antioxidant activity reported to be present in *Cleome* species. Hence, from the overview of the present study, hepatoprotective activity exhibited by methanolic leaves extract of *C. felina* might be due to the presence of active phyto-metabolites, such as 1,6-Anhydro-β-d-talopyranose, n-Hexadecanoic acid, L-Cystein S-sulfate, and other examined compounds, detected in methanolic leaves extract of *C. felina* analyzed by GCMS; the conclusion of the results can be drawn that *C. felina* leaves possess a novel hepatoprotective activity, and disputes its richness of phyto-compounds illustrated by functional group, elements, and phyto-compounds identification. Results of the analysis specify the abundance of *C. felina* in bioactive compounds, which could be alternatively used in the pharmacy field for isolation and preparation of eco-friendly drugs and would be a great scope for future hepatoprotective drug designing and an alternate resource as that of Silymarin.

## 4. Materials and Methods

### 4.1. Collection and Preparation of Plant Material

Wild *Cleome felina* L.f. plant was collected from the mountains of Darikonur (17.0268155 N, 75.3761952 E) village, Taluk-Jat, District-Sangli, Maharashtra, India. The plant specimens were authenticated and identified by Dr. Manoj M. Lekhak, professor (assistant) of the Department of Botany, Shivaji University, Kolhapur. The research plant herbarium was deposited at the Shivaji University, Kolhapur, labeled with the voucher specimen number HYS-02. The collected *C. felina* plant material (leaves, stem, and root) was washed under running tap water and shade-dried for 30–35 days. Each dried part of the *C. felina* was separately made into powder form using an electric blender and was stored in airtight containers at 4 °C and used for further analysis.

The 20 g powder of leaves, stem, and root was extracted with 250 mL of different solvents (acetone, methanol, and water) in the range of increasing polarity with its specific boiling temperature by a Soxhlet apparatus for 8–10 h. Then, the obtained extracts were filtered and made solvent-free under vacuum pressure. The obtained extracts were used for further analysis.

### 4.2. The Percentage Yield of Different Solvent Extracts

It was calculated as the total amount of the extract obtained, divided by the total amount of the powder of the plant samples. All the percentage yield of extract was repeated thrice and calculated by the following equation.

$$\mathrm{Yield}\;(\%)=\frac{weight\;of\;extract-weight\;of\;solvent\;free\;extract}{Total\;weight\;of\;powder}\times 100$$

### 4.3. Preliminary Screening of Phytocompounds for Different Solvent Extracts of C. felina

#### 4.3.1. Tests for Carbohydrates

##### Fehling’s Test

An equal volume of Fehling’s solution (A and B) was mixed and was added drop-wise to each heated extract solution. Brick-red precipitation was observed, which indicates the presence of reducing sugars [50].

#### 4.3.2. Test for Proteins

##### Biuret Test

A total of 1 mL of NaOH (10%) solution was combined with each extract and heated. To this, a few drops of CuSO_4_ (0.7%) solution was added, and the presence of proteins was indicated by the formation of a purplish-violet color [51].

#### 4.3.3. Test for Amino Acids

##### Ninhydrin Test

To each extract (2 mL), 2–5 drops of Ninhydrin solution (2%) was added and incubated for 1–2 min in a boiling water bath. The formation of purple color indicated the presence of amino acids [51].

#### 4.3.4. Test for Glycosides

##### Salkowski’s Test

A total of 1 mL of extract was combined with chloroform (2 mL), followed by treatment with concentrated H_2_SO_4_, and was then gently shaken. The presence of a steroidal ring was determined by the formation of a reddish-brown color [52].

#### 4.3.5. Test for Cardiac Glycosides

##### Cardiac Glycoside (Keller–Kiliani Test)

Plant extract (2 mL) was mixed with 5 mL distilled water and was shaken, followed by the addition of 2 mL glacial acetic acid (prepared by the addition of a few drops of ferric chloride). To this, 1 mL H_2_SO_4_ was added by the sides of the test tube. It was then observed for brown ring formation at the interface that shows the occurrence of cardiac glycosides, and also the formation of a violet ring might be noticed below the brown ring [53].

#### 4.3.6. Test for Phenols

##### Ferric Chloride Test

Test extracts were diluted in distilled water (3 mL) and reacted with ferric chloride (5% aqueous solution). A deep-blue or black color shows the positivity of phenols [51].

#### 4.3.7. Test for Flavonoids

##### Alkaline Reagent Test

Plant extracts were combined with 2% of NaOH solution (2 mL), which resulted in a deep-yellow color, which became colorless upon the addition of diluted acid (a few drops). This determines the presence of flavonoids [52].

#### 4.3.8. Test for Saponins

A total of 2 mL of plant extract was added to 10 mL distilled water and then shaken vigorously to obtain stable forth that indicates the presence of Saponins [53].

#### 4.3.9. Test for Terpenoids

##### Salkowski’s Test

Test extract (2 mL) was added to a test tube containing chloroform (2 mL), and they were thoroughly shaken to homogenize them. To this, 2 mL concentrated H_2_SO_4_ was added by the side of the test tube; the presence of terpenoids was indicated by the formation of a reddish-brown ring at the interface [53].

#### 4.3.10. Anthraquinones Glycosides

##### Borntrager’s Test

The sample extracts were shaken vigorously with a benzene layer and separated. To this, 10% ammonia solution was added in half the taken volume of the benzene layer. In the ammoniacal phase, pink, red, or violet color was observed for the anthraquinones glycosides presence [54].

#### 4.3.11. Test for Tannins

A total of 10 mL of distilled water was mixed with each extract and then filtered. To this, 5% ferric chloride (few drops) was added. The presence of tannins was determined by the formation of black or blue–green coloration or precipitation [53].

#### 4.3.12. Test for Alkaloids

A total of 2 mL of each extract was individually treated with 5 mL of hydrochloric acid (1.5% *v*/*v*) and filtered. The obtained filtrates were reacted with Mayer’s reagent (mercuric chloride (1.36 g) and potassium iodide (5 g) prepared in 100 mL distilled water). The presence of alkaloid was determined by the observation of yellow cream precipitation [51].

#### 4.3.13. Test for Betacyanins

Test extracts were reacted with 2 N NaOH and heated at 100 °C for 5 min; the occurrence of yellow color indicates the presence of betacyanins [55].

#### 4.3.14. Test for Quinone’s

An equal volume of extract was treated with concentrated H_2_SO_4_ and observed for the red color indicating the presence of quinone’s [51].

### 4.4. Fourier-Transform Infrared Spectroscopy (FT-IR) and Energy-Dispersive X-ray Spectroscopy (EDX) Analysis

The NICOLET 67000 FTIR spectrophotometer (NICOLET, Thermo Fisher Scientific, Waltham, MA, USA) was used for functional group identification. The obtained methanolic extract of leaves was dried under vacuum pressure for complete removal of moisture, grounded with crystals of potassium bromide, and pressed between guides by applying a vacuum to make the thin discs. Then, the spectrum of the extract was recorded in the transmittance mode between 400 cm^−1^ and 4000 cm^−1^ at a resolution of 4 cm^−1^. An elemental analysis of leaves powder was carried out to determine the elemental composition. The mass % of elements was determined by generating the spectra of the various micro and macro mineral elements using energy-dispersive X-ray spectroscopy (EDX) (JSM-IT 500LA, Tokyo, Japan) [19].

### 4.5. Phytochemical Determination of C. felina Methanolic Leaves Extract by Gas Chromatography–Mass Spectrometry (GCMS) Analysis

GCMS analysis of extract was performed using an Agilent 8890 MS gas chromatograph coupled to an Agilent 5977B Mass spectrometer detector, Palo Alto, CA, USA (MSD). Compounds were separated on a fused silica capillary column with a column dimension 30 m × 250 μm × 0.25 μm. The temperature of the injector was 250 °C, and 1 μL of the sample was injected in the split mode with a split ratio of 15:1. Helium (He) was used as a carrier gas, and the flow rate of the gas was 3 mL/min. The temperature program was as follows: initial temperature of 75 °C held for 0.5 min, followed by the ramping up of the temperature at a rate of 5 °C/min up to 300 °C, which was held for 20 min. The temperature of the MSD transfer line was 280 °C. For mass spectra determination, the MSD was operated in electron ionization (EI) mode, with ionization energy of 70 eV, while the mass range scanned was 50 *m*/*z*. The temperature of the ion source was 230 °C, and that of the MS quadrupole was 150 °C. The name, molecular weight, and structure of the components of the test materials were ascertained by comparing the mass spectra with the known compounds using an automated library search on the NIST MS Search program (version 2.3 accessed on 13 April 2023).

### 4.6. Antimicrobial Activity of C. felina Methanolic Leaves Extract

The *C. felina* methanolic leaves extract was analyzed for antimicrobial activity against selected pathogenic organisms by the agar well diffusion method in triplicates, according to the method of Rudrappa et al. [56]. The respective two Gram-positive bacteria: *S. aureus* (MTCC 6908) and *B. cereus* (MTCC 11778); two Gram-negative bacteria: *E. coli* (MTCC 40), *P. aeruginosa* (MTCC 9027); two yeast strains: *C. albicans* (MTCC 227) and *C. glabrata* (MTCC 3019) organisms were analyzed, which were procured from IMTECH, Chandigarh, India. The leaves methanolic (99%) extract of 100 mg/mL was prepared by dissolving in Dimethyl-sulfoxide DMSO (99%). Pathogenic organisms to be screened were cultured with a loopful of inoculation loop in Muller–Hinton broth (Himedia, Pvt Ltd, Mumbai, India), which was then incubated at 37 °C overnight. Pre-cultured pathogenic organisms were swabbed on separate nutrient agar plates in 0.5 McFarland concentrations, and a sterilized cork borer was used to produce 6 mm wells on cultured nutrient plates. For each organism, the respective wells were loaded with 100 µL of positive control (streptomycin for bacteria and nystatin for yeast, 100 µg/mL of each); 100 µL of negative control (DMSO); and 25, 50, 75, and 100 µL of *C. felina* methanolic leaves extract (100 mg/mL). All cultured plates were incubated at 37 °C for 24 h, and the zone of inhibition was recorded after the incubation period.

### 4.7. Experiment Animals

The male and female Wister albino rats (200–250 g) were facilitated from a central animal facility present in H.S.K. College of Pharmacy and Research Centre, Bagalkot. The experimental rats were maintained in a controlled environment of room temperature (22–28 °C), followed by relative humidity (65 ± 10%) and a light–dark cycle of 12 h. They were under observation in accordance to the guidelines of the Institutional Animal Ethics Committee (IAEC) and were provided with standard laboratory feed (Amruth, Sangli, Maharashtra, India) and were in continuous access to water as needed. Ethical approval was acquired from the Institutional Animal Ethics Committee (IAEC) of Hangal Shri Kumareshwar College of Pharmacy, Bagalkot-587101, Karnataka, India, with reference number HSKCP/IAEC, Clear/1/2022-23/R&D/ KUD 02.

### 4.8. Acute Toxicity of C. felina Methanolic Leaves Extract

The acute toxicity of Swiss albino mice was examined in accordance with the Organization for Economic Co-operation and Development (OECD) guidelines 425, by administering a single dose of *C. felina* methanolic leaves extract orally at concentrations of 84, 80, and 70 mg to the body weight (42, 40, and 35 g). The limit dose given per oral administration was 2000 mg/kg. The mice were closely monitored for 4 h for behavioral and toxicological signs. Subsequent observations continued for 14 days. The observations were made for various parameters, such as changes in the skin, fur, eyes, and mucous membranes; special attention was given to tremors, convulsions, salivation, diarrhea, lethargy, sleep, and coma. The determination of screening doses for the selected extract to evaluate their hepatoprotective activity was based on the results obtained from this study.

### 4.9. Paracetamol-Induced Hepatoprotective Activity by C. felina Methanolic Leaves Extract in Wister Albino Rats

The Wister albino rats were grouped into five sets, each consisting of four animals. In group 1 (Normal), rats were given a normal saline solution (vehicle) at a dosage of 10 mL/kg b.wt.; group 2 (Control) rats were treated with Paracetamol (PC), commonly known as *N*-acetyl-p-aminophenol (acetaminophen). Group 3 (standard) rats received Silymarine 20 mg/kg b.wt. Groups 4 and 5 were administered with *C. felina* methanolic leaves extract at a 50 and 100 mg/kg b.wt. dose, respectively, for 12 days duration via oral administration. All the group rats were sacrificed at the end of the experiment after 24 h of the last dose administration. Serum blood and liver tissue homogenates from all the experimental animals were collected and analyzed for biochemical markers and in vivo antioxidant enzyme activities.

#### 4.9.1. Biochemical Estimation in Blood Serum

After 24 h intervals of the final administration of PC, serum blood samples were obtained from each animal via the tail vein. The collected blood samples were then subjected to centrifugation at 3000 rpm for 10 min in order to separate the serum. The separated serum was utilized for the measurement of aspartate transaminase (AST), alanine transaminase (ALT), alkaline phosphatase (ALP), Total Bilirubin, and Total cholesterol levels by employing the described experiments of Iqbal et al. [57], using standard Biochemical diagnostic kits BIO-LA-TEST (Erbamannheim, Transasia Bio-Medicals Pvt Ltd., Mumbai, India).

##### Assessment of Aspartate Transaminase or Serum Glutamic Oxaloacetic Transaminase (AST/SGOT)

AST activity was estimated by following the method of Iqbal et al. [57], with minor adjustments. This assay was determined by preparing a fresh working reagent by combining 4 volumes of Reagent 1 with 1 volume of Reagent 2. To establish a baseline, a blank was analyzed using distilled water. For the test samples, 50 µL of serum was mixed with 500 µL of the working reagent at a temperature of 37 °C. The oxidation rate of NADH was measured kinetically by tracking the decrease in absorbance at 340 nm using an Auto analyzer (CPC Stat Fax 3000 Plus, Shimadzu, Kyoto, Japan).

##### Assessment of Alanine Transaminase or Serum Glutamic Pyruvic Transaminase (ALT/SGTP)

ALT activity was measured by employing the described procedure of Iqbal et al. [57], with a few concentration alterations. A working reagent was prepared by mixing Reagent with Reagent 2 to perform the estimation. The blank was analyzed using distilled water as a reference. For the test sample, 50 µL of serum was mixed with 500 µL of the working reagent at a temperature of 37 °C. The rate of NADH oxidation was determined kinetically by observing the decrease in absorbance at 340 nm using Auto analyzer (CPC Stat Fax 3000 Plus, Shimadzu, Kyoto, Japan).

##### Assessment of Alkaline Phosphatase (ALP)

ALP activity level was determined using Tietz et al. [58] method, with a few alterations. To determine the level of ALP activities, 20 µL of serum was combined with 1000 µL of ALP reagent. The ALP reagent consisted of Reagent 1. The change in absorbance caused by the formation of the yellow color was measured kinetically at 405 nm using an Auto analyzer (CPC Stat Fax 3000 Plus, Shimadzu, Kyoto, Japan). The magnitude of the absorbance change was directly proportional to the ALP activity present in the sample.

##### Assessment of Total Bilirubin

The estimation of Total Bilirubin was conducted using the Diazo method developed by Pearlman and Lee [59]. In this method, the working reagent used for this process was a combination of two components. Reagent 1 was the total Bilirubin reagent, and Reagent 2 was the sodium nitrite reagent (sodium nitrite). The analysis was carried out by preparing a blank solution consisting of 500 µL of the working reagent mixed with 25 µL of distilled water. Following this, the test sample is prepared by combining 500 µL of the working reagent with 25 µL of the test serum. The mixture was thoroughly mixed and then incubated for 5 min at 37 °C. The absorbance of the test sample was measured at a wavelength of 546 nm using an Auto analyzer (CPC Stat Fax 3000 Plus, Shimadzu, Kyoto, Japan).

##### Assessment of Total Cholesterol

Total cholesterol assessment was performed using the conducted experiment of Iqbal et al. [57], with some modifications. The assay was conducted using the PEG-CHOD-PAP (Polyethylene glycol-cholesterol oxidase-peroxidase-4- aminoantipyrine) method as an endpoint assay, incorporating the Lipid Clearing Factor (LCF). The standard was prepared by combining 100 µL of Reagent 1 with 10 µL of Reagent 2, namely, the Cholesterol standard containing cholesterol, preservatives, and stabilizers. To analyze the test sample, 1000 µL of the enzyme reagent is mixed with 10 µL of the sample and incubated at 37 °C for 10 min. The analysis was carried out using the reagent blank, followed by the standard and the test sample. The absorbance of the colored dye was measured at 505 nm using an Auto analyzer (CPC Stat Fax 3000 Plus, Shimadzu, Kyoto, Japan).

#### 4.9.2. In Vivo Antioxidant Estimation of Hepatic Tissues

In vivo antioxidant estimation of hepatic tissues was assessed. First, blood samples were collected from each animal through the tail vein using sterile centrifuge tubes containing heparin. Subsequently, all groups of rats were sacrificed, and their livers were removed. The excised livers were then rinsed with ice-cold normal saline to eliminate any remaining blood constituents. After rinsing, the livers were weighed and homogenized in cold phosphate buffer (0.1 M, pH 7.4). To obtain the post-mitochondrial supernatant (PMS), the tubes containing the liver homogenates were placed in a refrigerated centrifuge (high speed brushless centrifuge, MPW-350R (Shimadzu, Kyoto, Japan) and spun at 10,000 rpm for 10 min at 4 °C. The supernatant obtained from this step was further centrifuged at 17,000 rpm for 1 h at 4 °C. The resulting supernatant was used to evaluate liver function, reduced glutathione (GSH), catalase (CAT), superoxide dismutase (SOD), and lipid peroxidation (LPO).

##### Assay for Glutathione

Reduced glutathione was examined by the described method of Moron et al. [60], with some modifications. The preparation of reagents involves the following steps. A solution of 0.6 mM 5,5’-dithiobis (2-nitrobenzoic acid) (DTNB) was made by dissolving 0.00238 g of DTNB in 10 mL of methanol. For the phosphate buffer solution (PBS) with a pH 8, 9.4 mL of potassium hydrogen phosphate and 3 mL of potassium di-hydrogen phosphate are combined. Next, 1 mL of tissue homogenate was mixed with 6 mL of PBS at pH 8 and 1 mL of 0.6 mM DTNB. This mixture was then incubated at room temperature for 10 min, after which the absorbance was measured at 412 nm using an analyzer (CPC Stat Fax 3000 Plus, Shimadzu, Kyoto, Japan) against appropriate blank samples. To determine the glutathione content, a standard plot was used under the same experimental conditions.

##### Assay for Catalase

Catalase activity was assessed by employing the Claiborne method of Zeashan et al. [61], with minor changes. In summary, the assay mixture comprised 50 mM Phosphate Buffered Saline (PBS) at pH 7. To create a solution of 0.7 mM H_2_O_2_, 0.160 mL of H_2_O_2_ was combined with 100 mL of PBS. Following this, 1.95 mL of PBS at pH 7, 1 mL of H_2_O_2_, and 50 units of tissue homogenate were added together. Alterations in absorbance were measured at 240 nm using an auto analyzer (CPC Stat Fax 3000 Plus, Shimadzu, Kyoto, Japan) both at 0 min and 1 min. Catalase activity was determined in units per milligram of protein.

##### Assay for Superoxide Dismutase

Superoxide dismutase assay was determined using the procedure of Natikar et al. [62]. The sodium carbonate buffer, 0.5 N HCl, and epinephrine solution were prepared during the analysis. To initiate the experiment, combine 0.8 g of sodium carbonate buffer with 0.1 mL of tissue homogenate. Subsequently, the epinephrine solution was put into the quartz cuvettes. Another mixture of 0.8 g of sodium carbonate buffer and 0.1 mL of tissue homogenate was added to a separate cuvette. Absorbance was recorded at 295 nm using auto analyzer (CPC Stat Fax 3000 Plus, Shimadzu, Kyoto, Japan) both at 0 min and 1 min.

##### Assay for Lipid Peroxidation

The LPO reaction was determined by following the Buege and Aust [63] procedure with a few changes. To estimate the presence of thiobarbituric acid reactive substances (TBARS) in the tissue homogenate, 500 µL of 10% tissue homogenate was combined with 300 µL of 15% trichloroacetic acid (TCA), 300 µL of 0.375% thiobarbituric acid (TBA), and 30 µL of 5 N HCl. This mixture was then incubated at 95 °C for 15 min using a hot water bath. After cooling, the mixture was centrifuged at 2000 rpm, and the absorbance of the resulting supernatant was measured at 535 nm against an appropriate blank.

### 4.10. Statistical Analysis

All values were expressed as mean ± SEM. Results were analyzed statistically by using One-Way Analysis of Variance (ANOVA), by using Duncan’s multiple range test (DMRT) using IBM SPSS Statistics software version 20, followed by multiple Dunnett’s test (GraphPad Prism 10). The *p* < 0.05 was considered as significant. The effects of different treated groups were compared with that of normal groups.

## 5. Conclusions

The results of the current study on *C. felina* reports for the occurrence of remarkable secondary metabolites, such as glycosides, phenolics, flavonoids, saponins, terpenoids, and a few more. The fatal functional groups, like alcohols, amine salt, conjugated alkenes, sulfonyl chloride, halo compounds and major elements, like carbon, oxygen, magnesium, aluminum, silicon, phosphorus, sulfur, chlorine, potassium, and calcium, were revealed by FTIR and EDX spectroscopy analysis. Furthermore, the methanolic leaves extract exhibited the nutritional and pharmacological active phyto-compounds being detected as 1-Butanol, 3-methyl-, formate, 1-Decanol, 2-ethyl, 1,6-Anhydro-β-d-talopyranoseEthene, 1,2-bis(methylthio)-, Decane, 3-Methylene-,11-dimethyl-1-dodecene, AmLexanox, 1,2,3,4-Cyclopentanetetrol, (1α,2β,3β,4α), L-Cysteine S-sulfate, n-Hexadecanoic acid, and Flucarbazone screened by the GCMS technique. Despite phytochemical constitutions, methanolic leaves extract depicted potent antimicrobial, acute oral toxicity, and paracetamol-induced hepatoprotective activity. The exhibition of antimicrobial and hepatoprotective activity might be due to the 1, 6-Anhydro-β-d-talopyranose and n-Hexadecanoic acid, as these compounds were reported for their pharmacological applications. Overall, from the present data of the study, it can be concluded that *C. felina* leaves would be immense bedrock for the isolation of active phytochemicals, can be a future scope for treating microbial and hepatotoxic diseases, and could be utilized in numerous cosmetics, herbicides, and food industries. Future study is required for isolation and analysis of active phyto-components commencements for the antimicrobial and hepatoprotective activity of the *C. felina* leaves.

## Figures and Tables

**Table 1:** Different solvent extract yields of *C. felina* leaves, stem, and root.

Solvent	Extract Value g/100 g		
	Leaves	Stem	Root
Acetone	3.65 ± 0.03 ^g^	1.77 ± 0.01 ^h^	1.4 ± 0.03 ^i^
Methanol	17.01 ± 0.33 ^a^	9.63 ± 0.01 ^e^	8.4 ± 0.03 ^f^
Water	13.75 ± 0.01 ^c^	15.64 ± 0.02 ^b^	12.3 ± 0.03 ^d^

Data are mean ± standard error (n = 3). Different letters in superscript indicate significant difference (*p* < 0.05).

**Table 2:** Qualitative phytochemical screening of different solvent extracts of *C. felina* leaves, stem, and root.

Test Name	Leaves			Stem			Root		
	A	M	W	A	M	W	A	M	W
**Carbohydrates**									
Fehling’s test	+	+	+	+	+	−	+	−	−
**Protein**									
Biuret test	−	−	+	−	+	+	+	+	+
**Amino acids**									
Ninhydrin test	−	+	−	−	+	+	+	+	+
**Glycosides**									
Salkowski’s test	−	+	−	+	−	−	−	+	−
**Cardiac glycoside**									
Keller–Kiliani test	+	+	−	+	+	+	+	−	−
**Phenolics**									
Ferric chloride test	+	+	+	−	−	+	−	−	+
**Flavonoids**									
Alkaline reagent test	+	+	+	+	+	−	−	+	−
**Saponins**									
Froth Test	−	+	+	+	+	+	−	−	+
**Terpenoids**									
Salkowski’s test	−	+	−	+	−	−	−	+	−
**Anthraquinones glycosides**									
Borntrager’s test	+	+	+	+	+	+	−	−	+
**Tannins**									
Ferric chloride test	+	+	−	+	−	−	−	−	−
**Alkaloids**									
Mayer’s test	−	+	−	−	+	+	−	+	+
Wagner’s test	−	−	+	−	−	−	−	+	−
**Betacyanins**									
Sodium hydroxide test	−	−	+	+	+	+	−	+	−
**Quinones**	−	+	+	+	+	−	+	+	−

**Note**: A = Acetone, M = Methanol, W = Water, “+” = Present, and “−” = Absent.

**Table 3:** FT-IR peak values interpretation for bioactive functional groups of *C. felina* methanolic leaves extract.

Wavenumber (cm^−1^)	Intensities of Functional Group	Functional Group	Compound Class
3383.67	Strong, broad	O-H stretching	Alcohols
2920.81	Strong	N-H stretching	Amine salts
2850.61	Strong	N-H stretching	Amine salts
1627.68	Medium	C=C stretching	Conjugated alkene
1384.43	Strong	S=O stretching	Sulfonyl chloride
1176.30	Strong	C-O stretching	Tertiary alcohol
1072.47	Medium	C-N stretching	Amines
824.86	Strong	C-Cl stretching	Halo compounds
624.85	Strong	C-Br stretching	Halo compounds

**Table 4:** Element composition of *C. felina* leaves.

Serial No.	Elements	Mass %
		Leaves
1	C	36.48 ± 0.08
2	O	39.74 ± 0.19
3	Na	ND
4	Mg	1.80 ± 0.02
5	Al	0.35 ± 0.01
6	Si	0.46 ± 0.01
7	P	0.58 ± 0.01
8	S	1.01 ± 0.01
9	Cl	2.15 ± 0.02
10	K	2.031 ± 0.02
11	Ca	15.13 ± 0.06
Total		100.00

Note: ND- Not determined.

**Table 5:** Chemical composition of *C. felina* methanolic leaves extract screened by GCMS analysis.

Retention Time	Area	Identified Compound Name	Area (%)	Molecular Formula	Molecular Weight
3.669	76,453.684	Ethene, 1,2-bis(methylthio)-	7.21	C_4_H_8_S_2_	120.2 g/mol
4.146	42,618.846	Decane	4.02	C_10_H_22_	142.28 g/mol
4.919	16,409.742	Flucarbazone	1.55	C_12_H_11_F_3_N_4_O_6_S	396.30 g/mol
5.808	516,902.062	1-Butanol, 3-methyl-, formate	48.79	C_6_H_12_O_2_	116.16 g/mol
5.973	141,915.824	1-Decanol, 2-ethyl-	13.40	C_12_H_26_O	186.33 g/mol
6.148	21,971.905	1,2,3,4-Cyclopentanetetrol, (1α,2β,3β,4α)	2.07	C_5_H_10_O_4_	134.13 g/mol
6.937	19,474.186	L-Cysteine S-sulfate	1.84	C_3_H_7_NO_5_S_2_	201.2 g/mol
14.746	132,340.738	1,6-Anhydro-β-d-talopyranose	12.49	C_6_H_10_O_5_	162.14 g/mol
23.045	32,613.550	3-Methylene-7,11-dimethyl-1-dodecene	3.08	C_15_H_28_	208.38 g/mol
26.373	17,986.273	n-Hexadecanoic acid	1.70	C_16_H_32_O_2_	256.42 g/mol
30.074	14,265.444	4-Bromo-2,6-difluorobenzyl alcohol	1.35	C_7_H_5_BrF_2_O	223.01 g/mol
37.405	26,479.282	Amlexanox	2.50	C_16_H_14_N_2_O_4_	298.29 g/mol
Total %			100.00		

**Table 6:** Antimicrobial activity of the *C. felina* methanolic leaves extract against Gram-positive, Gram-negative bacteria, and two yeast strains.

Organisms	Zone of Inhibition in mm					
	Positive Control	CFMLS Extract				Negative Control
	100 µL	25 µL	50 µL	75 µL	100 µL	100 µL
*S. aureus*	18.33 ± 11.66 ^cd^	ND	ND	8.67 ± 5.51 ^j^	12.3 ± 7.84 ^g^	ND
*B. cereus*	20.33 ± 12.94 ^a^	ND	ND	10.67 ± 6.78 ^i^	13.67 ± 8.69 ^f^	ND
*E. coli*	14.33 ± 9.12 ^ef^	ND	ND	7.67 ± 4.87 ^k^	11.33 ± 7.21 ^hi^	ND
*P. aeruginosa*	18.67 ± 11.87 ^bc^	ND	5.33 ± 3.39 ^l^	7.67 ± 4.87 ^k^	10.67 ± 6.78 ^i^	ND
*C. albicans*	19.33 ± 12.30 ^b^	ND	ND	11.67 ± 7.42 ^gh^	14.67 ± 9.33 ^e^	ND
*C. glabrata*	17.67 ± 11.24 ^d^	3.33 ± 2.12 ^m^	5.67 ± 3.60 ^l^	7.33 ± 4.66 ^k^	12.33 ± 7.84 ^g^	ND

Data are mean ± standard error (n = 3). Different letters in superscript indicate significant difference (*p* < 0.05); the same superscript letters indicates no significant difference (*p* < 0.05) between each other, and ND = not determined. CFMLS: *C. felina* methanolic leaves extract.

**Table 7:** The effect of *C. felina* methanolic leaves extract towards serum blood biochemical markers.

Groups	Treatment	AST (U/L)	ALT (U/L)	ALP (U/L)	Total Bilirubin (mg/dL)	Total Cholesterol (mg/dL)
Group-1 Normal	Vehicle	136.2 ± 0.40	76.20 ± 0.48	238.1 ± 1.19	0.48 ± 0.003	35.77 ± 0.38
Group-2 Control	Paracetamol (PC)	857.7 ± 2.65 ****	722.9 ± 3.20 ****	736.9 ± 0.67 ****	1.98 ± 0.00 ****	60.50 ± 1.12 ****
Group-3 Standard	Silymarin	127.3 ± 0.91 *	70.85 ± 0.85 *	224.0 ± 0.49 ****	0.45 ± 0.001 ***	33.13 ± 0.31 **
Group-4 Low dose	PC + CFMLS (50 mg/kg p. o)	220.5 ± 0.79 ****	47.18 ± 0.82 ****	203.1 ± 2.38 ****	0.21 ± 0.00 ****	38.46 ± 0.67 *
Group-5 High dose	PC + CFMLS (100 mg/kg p. o)	248.0 ± 5.30 ****	34.48 ± 0.48 ****	445.0 ± 1.45 ****	0.17 ± 0.002 ****	39.03 ± 0.41 **

All values of table presented as mean ± SEM, one-way Analysis of Variance (ANOVA), followed by multiple Dunnett’s test, * *p* < 0.05, ** *p* < 0.01, *** *p* < 0.001, and **** *p* < 0.0001 compared with normal group. (CFMLS: *C. felina* methanolic leaves extract).

**Table 8:** The effect of *C. felina* methanolic leaves extract towards in vivo antioxidant enzymes and lipid peroxidation.

Groups	Treatment	GSH (n mol/mg of Protein)	CAT (n mol/mg of Protein)	SOD (n mol/mg of Protein)	LPO (n mol/mg of Protein)
Group-1 Normal	Vehicle	100.8 ± 1.53	0.10 ± 0.00	337.2 ± 2.47	264.0 ± 17.48
Group-1 Control	Paracetamol (PC)	59.93 ± 0.30 **	0.02 ± 0.001 *	208.9 ± 7.57 ****	465.7 ± 28.54 ***
Group-1 Standard	Silymarin	346.4 ± 6.77 ****	0.93 ± 0.04 ****	353.9 ± 0.58 *	40.00 ± 8.13 ****
Group-1 Low dose	PC + CFMLS (50 mg/kg p.o)	51.29 ± 1.05 ***	0.18 ± 0.003 *	218.5 ± 00 ****	72.65 ± 2.41 ***
Group-1 High dose	PC + CFMLS (100 mg/kg p.o)	81.50 ± 3.80 *	0.17 ± 0.1 *	407.9 ± 6.16 ****	194.0 ± 0.59 *

All values of table presented as mean ± SEM, one-way Analysis of Variance (ANOVA), followed by multiple Dunnett’s test, * *p* < 0.05, ** *p* < 0.01, *** *p* < 0.001, and **** *p* < 0.0001 compared with normal group. (CFMLS: *C. felina* methanolic leaves extract).

## Data Availability

Data are available upon request.
